# A 12-Week, Randomized, Double-Blind, Placebo-Controlled Clinical Trial of *Gastrodia elata* Extract for Memory Support in Adults with Subjective Memory Complaints

**DOI:** 10.3390/ph19101558

**Published:** 2026-10-01

**Authors:** Ayusha Pandey, Dasol Park, Wonbae Ha, Chang Su Kim, Eun Suk Lee, Chan-Mi Park, Jungtae Leem, Kang-Beom Kwon

**Affiliations:** 1 Digital Healthcare, Graduate School of JABA, Wonkwang University, Iksan 54538, Jeonbuk State, Republic of Korea; ayushapandey232@gmail.com; 2 Department of Diagnostics, College of Korean Medicine, Wonkwang University, Iksan 54538, Jeonbuk State, Republic of Korea; mare927@naver.com; 3 Department of Korean Medicine Rehabilitation, College of Korean Medicine, Wonkwang University, Iksan 54538, Jeonbuk State, Republic of Korea; ha0530@gmail.com; 4 Jeonbuk State Agricultural Research & Extension Services, Iksan 54591, Jeonbuk State, Republic of Korea; florigen5329@korea.kr (C.S.K.); es10014@korea.kr (E.S.L.); 5 Department of Physiology, College of Korean Medicine, Wonkwang University, Iksan 54538, Jeonbuk State, Republic of Korea; cmhappy66@naver.com; 6 Department of Il-won Integrative Medicine, Wonkwang University Korean Medicine Hospital, Iksan 54538, Jeonbuk State, Republic of Korea; 7 Research Center of Traditional Korean Medicine, College of Korean Medicine, Wonkwang University, Iksan 54538, Jeonbuk State, Republic of Korea

**Keywords:** *Gastrodia elata*, cognitive dysfunction, memory disorders, randomized control trial, functional food

## Abstract

**Background/Objectives:** Subjective memory complaints (SMCs) represent an early window for cognitive intervention; however, no approved pharmacological treatment exists at this stage. *Gastrodia elata* is a traditional medicinal plant, and preclinical studies suggest that it may influence synaptic plasticity, neurotrophic signaling, and neuroinflammatory pathways involved in memory; however, human evidence remains limited. **Methods:** This study was a randomized, participant- and assessor-blinded, placebo-controlled clinical trial in which the Clinical Research Coordinator, statistician, physician, and participants remained unaware of group assignment throughout the study period. Eighty adults with normal objective cognitive function and subjective memory complaints were randomized; 76 participants were included in the full analysis set (36 *G. elata*, 40 placebo) and all 80 in the safety set. **Results:** The prespecified primary outcome showed a small between-group difference in MoCA-K total score change: *G. elata* 0.72 ± 1.34 vs. placebo 0.13 ± 1.07 (between-group *p* = 0.034). Exploratory secondary analyses showed significant differences in MoCA-K delayed recall, K-MMSE total, K-MMSE memory recall and BDNF, whereas Digit Span and IL-6 did not differ significantly between groups. No serious adverse events occurred. The trial was retrospectively registered in CRIS (KCT0011966) on 12 May 2026. **Conclusions:** These findings provide preliminary evidence that *G. elata* supplementation may support selected cognitive outcomes in adults with SMCs, although the clinical relevance requires confirmation in larger, prospectively registered trials. Further studies incorporating neuroimaging and prespecified biomarker analyses are warranted to determine whether the observed cognitive findings are accompanied by objective changes in brain function or circulating biomarkers.

## 1. Introduction

A decline in memory is a hallmark of cognitive aging and may occur even in the absence of a clear neurological disorder [1]. Subjective memory complaints (SMCs), which are self-perceived difficulties in memory not necessarily confirmed by objective testing, are commonly reported in older adults; however, growing evidence indicates that they are also prevalent among younger individuals [2]. Such experiences of forgetting, a core feature of SMCs, are now recognized as an important early warning sign rather than simply a normal consequence of aging [3]. Self-reported memory difficulties can occur even in young adults and can be triggered by stress, lifestyle, and early brain changes. These difficulties may serve as early warning signs of cognitive impairment that develops later in life [4]. Importantly, individuals often recognize these changes before they are detectable through standard clinical assessments, highlighting the value of SMCs as an early marker of decline [5,6]. Therefore, investigating memory function across the lifespan, particularly in younger populations, is essential for understanding early cognitive changes and identifying opportunities for timely intervention [7].

Addressing SMCs is clinically imperative because they represent the earliest symptomatic window of dementia risk, carry measurable biomarker evidence of preclinical Alzheimer’s pathology [8], and offer the optimal opportunity for modifiable risk factor intervention before irreversible neuronal loss occurs [9]. However, studies have largely failed to demonstrate robust or consistent evidence that pharmacological treatments can prevent dementia or slow cognitive decline, and no approved pharmacological intervention currently exists specifically for individuals at the SMC stage [10]. This therapeutic gap has shifted attention toward functional foods, which contain bioactive compounds that promote health by reducing chronic disease risk factors and supporting normal physiological functioning, including cognitive health [11]. Preclinical studies suggest that *G. elata* and gastrodin may influence pathways relevant to neuronal function; however, human clinical evidence remains limited and heterogeneous, and efficacy for subjective memory complaints has not been established [12,13].

*Gastrodia elata* is an orchidaceous medicinal plant known as Tianma in traditional Chinese medicine and has been used for centuries. Its dried rhizome has been widely applied in the management of neurological conditions [14]. The major bioactive component, gastrodin, exhibits multiple pharmacological effects, including antioxidant, neuroprotective, and cognitive-enhancing properties [15]. Building on this preclinical foundation, our previous clinical trial demonstrated that 12 weeks of *G. elata* supplementation significantly improved Alzheimer’s Disease Assessment Scale-cognitive subscale and Korean Mini-Mental State Examination (K-MMSE), elevated serum brain-derived neurotrophic factor (BDNF) and total antioxidant status with no serious adverse events (AEs) observed. These results support its favorable safety profile and potential as a functional food ingredient for cognitive decline prevention and management [13].

Therefore, the present study was designed to address this gap. Targeting adults with SMCs, this investigation employed the Korean version of the Montreal Cognitive Assessment (MoCA-K) as a primary outcome measure, which is sensitive to mild cognitive changes and early impairment. MoCA-K sub-scores, the Digit Span Test, K-MMSE, serum BDNF, and interleukin-6 (IL-6) were also incorporated as secondary outcome measures to better understand the underlying mechanisms of any observed effects. Both efficacy and safety were comprehensively evaluated. The primary aim of this study was to evaluate the efficacy and safety of 12 weeks of *G. elata* supplementation on memory function in adults with SMCs through a randomized, blinded, placebo-controlled design.

## 2. Results

### 2.1. Baseline Characteristics

**Figure 1:**
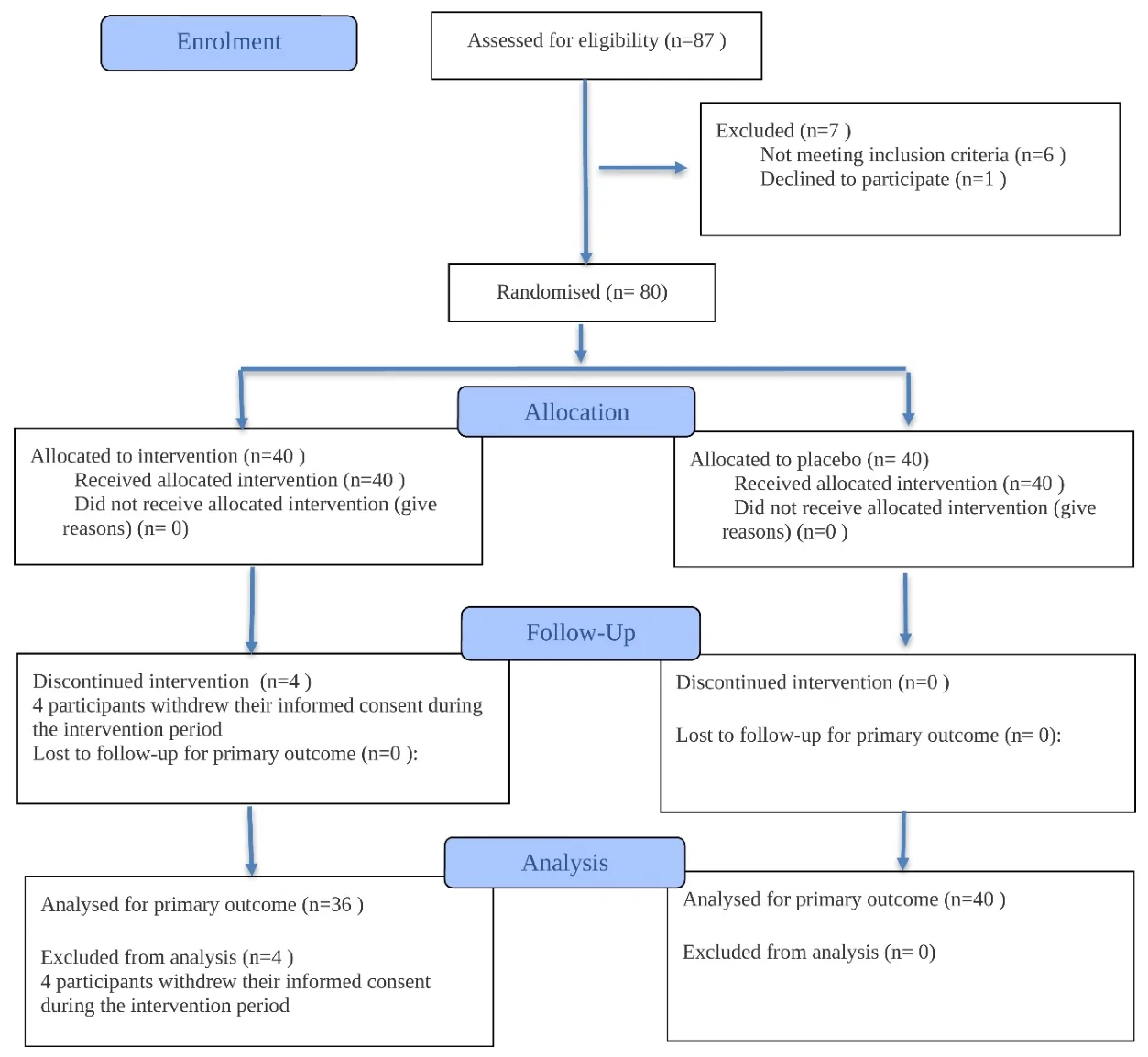
Flow of the study using CONSORT diagram. FAS, full analysis set; *G. elata*, *Gastrodia elata* Blume; SS, safety set.

The participants were enrolled between March and July 2025. Of 87 individuals screened, seven were excluded (six did not meet eligibility criteria and one withdrew consent before randomization). Eighty participants were randomized (40 per group). Four *G. elata* group participants withdrew consent after randomization and had no post-baseline efficacy assessment, leaving 76 participants in the FAS; all 80 participants were included in the safety set (Figure 1).

To confirm pre-treatment equivalence, baseline demographic and lifestyle characteristics, encompassing sex, age, education, height, weight, body mass index, blood pressure, alcohol consumption, and smoking habits, were assessed and compared between groups. No significant differences were detected between the two groups across any of these variables (*p* > 0.05) (Table 1).

### 2.2. Efficacy Outcomes

Analysis of efficacy outcomes demonstrated that 12 weeks of *G. elata* supplementation produced meaningful improvements in cognitive function compared with placebo.

#### 2.2.1. Primary Outcome

At baseline, MoCA-K total scores were 27.86 ± 1.40 in the *G. elata* group and 28.25 ± 1.33 in the placebo group. Following 12 weeks of intervention, the *G. elata* group demonstrated a significantly greater increase in MoCA-K total score compared to placebo (0.72 ± 1.34 vs. 0.13 ± 1.07 points, respectively; *p* = 0.034; Table 2).

#### 2.2.2. Secondary Outcome

At baseline, delayed recall scores were 4.00 ± 0.99 in the *G. elata* group and 4.50 ± 0.91 in the placebo group. Within the MoCA-K, the delayed recall test showed a significantly greater improvement in the *G. elata* group (0.64 ± 1.10 points) compared with a marginal decline in the placebo group (−0.10 ± 0.96 points), with between-group differences remaining significant after covariate adjustment (ANCOVA-adjusted *p* = 0.0003). No significant between-group differences were observed for Digit Span Forward or Backward performance (ANCOVA-adjusted *p* = 0.132 and *p* = 0.888, respectively). At baseline, K-MMSE total scores were 28.50 ± 1.32 in the *G. elata* group and 29.10 ± 0.96 in the placebo group. After 12 weeks, the *G. elata* group demonstrated a significantly greater increase in K-MMSE total score (0.94 ± 1.33 points) relative to the placebo group (0.28 ± 1.04 points), with differences remaining significant following covariate adjustment (ANCOVA-adjusted *p* = 0.003). Consistently, K-MMSE memory recall scores, for which baseline values were 2.06 ± 1.04 in the *G. elata* group and 2.60 ± 0.74 in the placebo group, showed a greater increase in the *G. elata* group (0.72 ± 1.09 points) than in the placebo group (0.23 ± 0.73 points), with between-group differences retained after adjustment (ANCOVA-adjusted *p* = 0.004). Following intervention, serum BDNF increased in the *G. elata* group (38,676.53 to 40,016.67 pg/mL) but decreased in the placebo group (39,820.50 to 37,622.18 pg/mL), with the between-group difference in change scores reaching significance (*p* = 0.026). In contrast, IL-6 levels did not differ significantly between the two groups throughout the study period (*p* > 0.05) (Table 2).

Unadjusted between-group *p* values were derived from independent *t* tests of change scores. For outcomes with significant baseline imbalance, ANCOVA was additionally performed using the corresponding baseline outcome value as a covariate. The adjusted *p* values were 0.0003 for MoCA-K delayed recall, 0.132 for Digit Span Forward, 0.003 for K-MMSE total, and 0.004 for K-MMSE memory recall. All other *p* values in Table 2 are unadjusted unless explicitly marked.

### 2.3. Safety and AEs

The safety set included all 80 participants. Two participants in the *G. elata* group experienced three events (myalgia, indigestion, and acute rhinitis), and one placebo participant experienced one event (shoulder pain), corresponding to 5.0% and 2.5% of participants, respectively.

All events were graded as mild and assessed as unrelated or clearly unrelated to the study product; no serious adverse events occurred. Mean adherence was 94.19 ± 5.79% in the *G. elata* group and 95.78 ± 5.05% in the placebo group, and no participant was excluded for adherence below 80%. Between-group differences in hemoglobin and mean corpuscular hemoglobin concentration remained within reference ranges, and no clinically meaningful laboratory, vital-sign, anthropometric, or electrocardiographic abnormality was identified. Detailed results of the safety-related laboratory assessments, including hematology, blood chemistry, and urinalysis, are presented in Supplementary Table S1.

## 3. Discussion

Treatment with *G. elata* resulted in significantly greater improvements in both MoCA-K and K-MMSE total scores compared to placebo. Among the sub-domains evaluated, delayed recall on the MoCA-K and memory recall on the K-MMSE exhibited between-group differences. These behavioral findings were further supported by a favorable shift in serum BDNF concentrations within the treatment group, providing neurobiological support for the observed cognitive improvements. No serious AEs were documented throughout the study period. However, participants entered the trial with normal objective cognitive function and high baseline MoCA-K and K-MMSE scores. Several subdomains were at or near their scale maxima, limiting the measurable range for improvement. Accordingly, the observed between-group findings were concentrated in delayed-recall measures and should not be interpreted as evidence of broad cognitive enhancement. The results are best regarded as preliminary signals that require confirmation with instruments designed to minimize ceiling effects in cognitively normal populations.

Plant-derived nootropic agents constitute a longstanding therapeutic approach to enhancing cognitive capacity and neurological function [16]. The cognitive-restorative properties of nootropics function by increasing cerebral blood perfusion, facilitating cholinergic and glutamatergic neurotransmission, and suppressing monoamine oxidase enzymatic activity [17,18]. Within this mechanistic framework, *G. elata*, administered as a single botanical agent, may exert neurochemically targeted neuroprotection through multiple converging preclinical pathways identified in previous studies. The principal bioactive constituent, gastrodin (4-hydroxybenzyl alcohol 4-O-β-d-glucopyranoside), has been implicated in synaptic plasticity preservation via the cAMP/PKA/CREB and AKT1/NF-κB signaling cascades [19,20]. Complementary preclinical evidence further implicates GABAergic normalization, dopaminergic regulation, and adrenal steroid modulation in hippocampal and cortical regions as additional contributors to its memory-enhancing effects [21].

Previous randomized trials of *G. elata* have examined middle-aged or late-middle-aged adults using different products and cognitive instruments. The present study provides incremental evidence in adults aged 20–65 years with normal objective cognition but subjective memory complaints, using MoCA-K total score as the prespecified primary outcome and including recall subdomains, Digit Span, K-MMSE, BDNF, and IL-6 as secondary measures. These design differences do not establish superior efficacy or broad novelty; rather, they extend the limited human evidence to a distinct, younger population and outcome set. We employed the MoCA-K as a primary outcome measure, as this multidomain cognitive screening tool has been recognized for its broad construct coverage and validated sensitivity in capturing subtle cognitive changes in young and middle-aged adults (20–65 years) presenting with SMCs. In a parallel 12-week randomized, placebo-controlled trial enrolling middle-aged adults of 40–65 years, cognitive function was assessed using the Alzheimer’s Disease Assessment Scale-cognitive subscale, an instrument specifically developed to quantify cognitive dysfunction across memory, language, and praxis domains, and calibrated to detect meaningful change in populations with established or emerging cognitive deficits, with significant cognitive benefits reported for *G. elata* [13]. Taken together, both trials mark a meaningful transition in the research trajectory of *G. elata*, shifting from symptomatic management in clinically diagnosed dementia toward earlier, preventive intervention targeting individuals at the pre-clinical SMC stage. In contrast, a trial assessing *G. elata* in healthy late-middle-aged adults (50–64 years; K-MMSE > 24) using the Computerized Neuropsychological Test reported no significant between-group differences in verbal learning or the Stroop test in the full analysis set [22]. For serum BDNF, both the present study and the parallel trial in middle-aged adults reported significant between-group neurotrophin differences, suggesting neuroprotective effects on hippocampal memory consolidation. The absence of a significant serum BDNF change in the late-middle-aged cohort [22] is likely attributable to higher baseline neurotrophin levels in a cognitively healthy population. To our knowledge, the Digit Span Test has not previously been used as an outcome measure in clinical trials of *G. elata*, representing a novel contribution of the present study to characterizing the attentional dimension of its cognitive effects. IL-6 did not show a between-group difference. In a relatively young, objectively cognitively normal sample with low baseline inflammatory burden and a 12-week intervention, the absence of change is not unexpected; however, the study was not designed or powered to establish an anti-inflammatory mechanism. The between-group difference in BDNF change was exploratory. The within-group BDNF change was not statistically significant, and no correlation analysis between BDNF change and cognitive outcomes was prespecified or performed. Therefore, the biomarker finding does not demonstrate mediation or a neuroprotective mechanism and should be interpreted as hypothesis-generating only.

This study focused on primary prevention, distinguishing it from the dominant therapeutic framework in dementia research. Currently approved pharmacological agents (three cholinesterase inhibitors and memantine, the N-methyl-D-aspartate receptor antagonist) are restricted to symptomatic management in clinically established Alzheimer’s disease [23,24], by which time substantial and often irreversible neuronal loss has already occurred. Although anti-amyloid immunotherapies (lecanemab and donanemab) represent a mechanistic advance by consistently reducing cerebral amyloid burden in early-stage, their effect on clinical decline remains modest [25]. By focusing on the pre-clinical SMC stage, predominant early clinical manifestations of both amnestic Mild Cognitive Impairment and Alzheimer’s disease [26], as the intervention window, this study demonstrates meaningful memory improvements prior to the onset of overt cognitive decline. With approximately 50 million people currently living with dementia worldwide and the global prevalence projected to triple by 2050 [27], halting disease progression at this stage preserves quality of life and functional independence while reducing the financial and caregiving burdens that intensify with each advancing stage of cognitive decline [28]. Notably, societal costs of Alzheimer’s disease rise by at least 50% between consecutive severity levels, with informal caregiving comprising nearly half of total expenditure across all care settings [29], such that even a modest delay in disease progression translates into substantial economic savings and meaningful reduction in caregiver strain. The present trial did not enroll participants with mild cognitive impairment or Alzheimer’s disease and did not assess disease progression. It therefore cannot support conclusions regarding dementia prevention or modification of a neurodegenerative process. The findings are limited to short-term changes on cognitive screening measures in adults with normal objective cognition and subjective memory complaints.

This trial has several notable methodological strengths. First, the randomized, blinded, placebo-controlled design minimizes selection bias and the risk of expectation effects, providing a rigorous framework for causal inference. Further, the use of two independent cognitive assessment instruments (MoCA-K and K-MMSE) strengthens confidence in the observed cognitive improvements by demonstrating convergent validity across different measurement approaches. Notably, incorporation of serum BDNF as a biological correlate bridges the gap between clinical outcomes and neurobiological mechanisms, lending mechanistic plausibility to the behavioral findings. The application of ANCOVA to adjust for significant baseline imbalances across outcome variables represents a key analytical strength, ensuring that between-group comparisons reflect treatment effects rather than pre-existing differences. This study investigated *G. elata* in a cognitively healthy adult population with SMCs, positioning it explicitly within a primary prevention framework. The 20–65-year eligibility range was selected prospectively to examine subjective memory complaints across adulthood rather than age-related cognitive decline specifically. The resulting sample was relatively young (mean age 35.55 years), which limits extrapolation to older adults and populations at elevated dementia risk. No age-stratified analysis was prespecified or performed, and age-related heterogeneity remains a limitation.

There were other several limitations to this study. Retrospective registration prevented prospective public verification of the outcome hierarchy and analysis plan and therefore remains an important reporting limitation. However, the protocol and statistical framework had been documented in the IRB-approved study records before enrollment, and the protocol and clinical study report were later evaluated during MFDS regulatory review. Several statistical limitations should also be considered. The primary-outcome difference observed in this trial was smaller than the difference assumed for sample-size planning, reinforcing the preliminary nature of the findings. Four post-randomization withdrawals occurred only in the *G. elata* group, which may have introduced attrition bias. Baseline values were lower in the *G. elata* group for several outcomes that subsequently favored the active group; therefore, regression to the mean cannot be excluded, and the adjusted findings remain exploratory. In addition, MoCA-K and K-MMSE are bounded scales with pronounced ceiling effects in this sample; results from parametric analyses should therefore be interpreted cautiously. The primary-outcome difference observed in this trial was smaller than the difference assumed for sample-size planning, a discrepancy that reinforces the preliminary nature of the findings and underscores the need for confirmation in a larger trial. Also, the effectiveness of blinding was also not formally assessed, as participants, investigators, and outcome assessors were not asked to guess treatment allocation; consequently, successful blinding cannot be empirically confirmed. Furthermore, the study population was limited to Korean participants recruited from a single Korean Medicine Hospital, which may limit the generalizability of the findings to broader and more ethnically diverse populations. In addition, SMCs may arise from various underlying etiologies, contributing to within-group heterogeneity that was not fully characterized. Lifestyle-related factors that could independently influence cognitive function including sleep quality and psychosocial stress were not comprehensively controlled. Future studies should employ longer follow-up periods of at least 12 months to determine whether the cognitive benefits observed at 12 weeks are sustained beyond the intervention period. Multicenter trials with larger and more diverse study populations are needed to enhance the generalizability of the findings and validate the observed effects across different demographic groups. To mitigate the learning effects inherent in repeated MoCA-K administration, future trials should employ alternate validated parallel versions across consecutive assessment timepoints. Future trials incorporating structural magnetic resonance imaging or functional neuroimaging would provide objective, independent verification of whether G. elata supplementation induces measurable neurobiological change.

## 4. Materials and Methods

### 4.1. Trial Registration

This trial was retrospectively registered in the Clinical Research Information Service (CRIS; KCT0011966) on 12 May 2026, after completion of the clinical study report. The first participant was enrolled on 4 March 2025, and the final participant completed the final visit on 11 July 2025. Before participant enrollment, the study protocol, including the planned efficacy assessments and statistical methods, was reviewed and approved by the Institutional Review Board of Wonkwang University Korean Medicine Hospital (Iksan, Republic of Korea). After study completion, the protocol and clinical study report were submitted to the Korean Ministry of Food and Drug Safety (MFDS; Cheongju, Republic of Korea) as part of the regulatory review for product authorization, which has subsequently been granted.

### 4.2. Study Design

This study was a prospective, randomized, blinded, placebo-controlled clinical trial conducted over 12 weeks at a single-center Wonkwang University Korean Medicine Hospital to assess the efficacy and safety of *G. elata* Blume in improving memory among adults experiencing subjective memory decline. The study protocol and informed consent documents were reviewed and approved by the Institutional Review Board of Wonkwang University Korean Medicine Hospital (initial approval: 12 September 2024), and the trial adhered to the ethical principles outlined in the Declaration of Helsinki. Prior to initiation of any study-related procedures, written informed consent was obtained from all participants. During the screening phase, thorough assessment was performed to determine patient eligibility based on predefined inclusion and exclusion criteria. This assessment involved the collection of demographic data, medical and medication histories, alcohol and smoking habits, physical examination findings, vital signs, and anthropometric parameters including height, weight, and body mass index. Self-rated questionnaires were additionally administered to evaluate SMCs and Adult Attention-Deficit/Hyperactivity Disorder Self-Report Scale. Clinical laboratory assessments comprising hematology, blood chemistry, and urinalysis were performed alongside electrocardiography and urine pregnancy tests for women of childbearing age. Eligible participants were randomly assigned in a 1:1 ratio to either the *G. elata* or placebo group through a computer-generated block randomization schedule administered by Aidone Co., Ltd. (Seoul, Republic of Korea) which was also responsible for trial monitoring, data management, and statistical analysis for the study. The *G. elata* and placebo tablets were designed to match in appearance, final weight, packaging, and administration schedule, and treatment codes were not routinely released before database lock. However, the effectiveness of blinding was not formally assessed by asking participants, investigators, or outcome assessors to guess treatment allocation. Consequently, successful blinding cannot be empirically confirmed. Both the *G. elata* and placebo groups received identical 900 mg tablets that matched in appearance and weight administered orally once daily after breakfast for 12 consecutive weeks. Efficacy outcomes were evaluated at baseline and at the end of the intervention period using the MoCA-K, Digit Span Test, K-MMSE, serum BDNF, and IL-6. Safety was monitored throughout the trial by recording AEs and reviewing vital signs and laboratory test results (Table 3).

### 4.3. Eligibility Criteria

Participants who met the following criteria were eligible for the inclusion: (1) Adult men and women aged 20–65 years with a Subjective Memory Complaints Questionnaire score ≥ 1; (2) Individuals who did not meet the clinical diagnostic criteria for attention-deficit/hyperactivity disorder; (3) Individuals who did not meet the definition of dementia according to the Diagnostic and Statistical Manual of Mental Disorders; and (4) Individuals who voluntarily decided to participate after receiving detailed explanation of this human application test, fully understood the information, and provided written consent agreeing to comply with precautions. Participants who met any of the following criteria were excluded from the study: (1) Individuals with abnormal blood test findings, alanine aminotransferase or aspartate aminotransferase ≥ 3 times the upper limit of normal, hemoglobin ≤ 8 g/dL or platelets < 100,000/mm^3^, estimated glomerular filtration rate < 60 mL/min/1.73 m^2^; (2) patients with cognitive impairment due to brain disease or psychiatric disorders; (3) patients with uncontrolled hypertension (systolic blood pressure > 140 mmHg or diastolic blood pressure > 90 mmHg); (4) patients with uncontrolled diabetes (HbA1C > 7.0%); (5) individuals whose thyroid-stimulating hormone levels are outside the institutional normal range; (6) individuals with severe psychiatric disorders such as major depression, schizophrenia, alcohol dependence, or drug dependence; (7) individuals with a history of unstable angina, myocardial infarction, transient ischemic attack, or coronary intervention including coronary artery bypass surgery within 6 months prior to the consent date; (8) individuals with a history of severe head trauma accompanied by loss of consciousness within 6 months prior to the consent date; (9) individuals with a history of acute stroke within 3 months prior to the consent date; (10) patients with severe hearing or visual impairment that makes efficacy evaluation impossible; (11) pregnant or breastfeeding women, or individuals planning pregnancy; (12) individuals with allergies or adverse reactions to the test substance; (13) alcohol consumption [men ≥ 40 g/day, women ≥ 20 g/day (according to World Health Organization daily alcohol intake guidelines)]; (14) excessive smokers (≥20 cigarettes/day); (15) individuals who consumed health functional foods related to the target functionality within 3 months prior to screening; (16) individuals who participated in another human application test within 3 months prior to screening; and (17) individuals deemed inappropriate for study participation by the principal investigator due to diagnostic laboratory results or other reasons.

### 4.4. Interventions

Naturalway Co., Ltd. (Pocheon, Republic of Korea) manufactured both the *G. elata* tablets and corresponding placebo.

#### 4.4.1. *Gastrodia Elata* Blume Tablet Group

**Figure 2:**
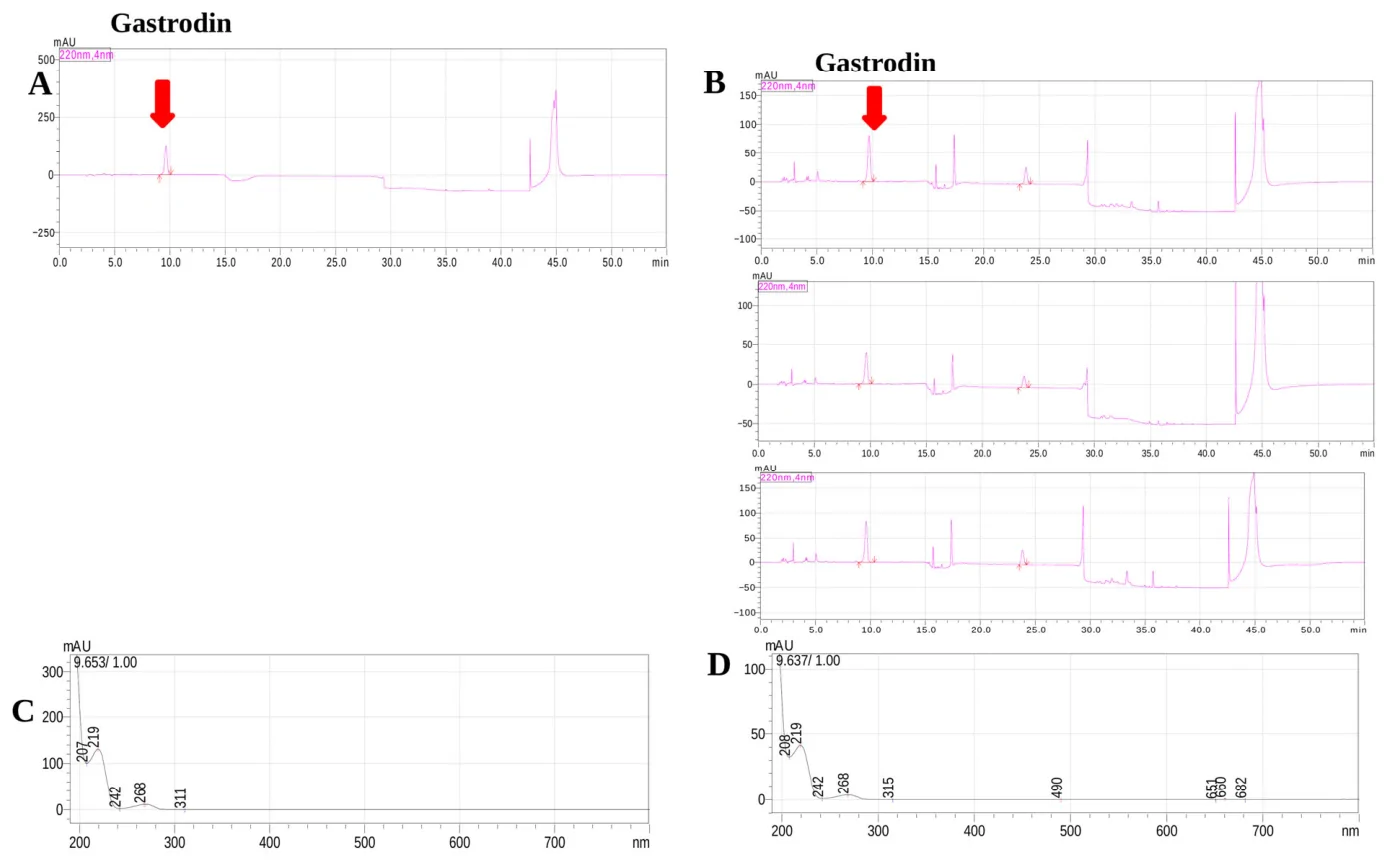
High-performance liquid chromatography (HPLC) chromatogram and spectrum. Each active tablet had a final weight of 900 mg and contained 500 mg of *G. elata* extract (55.556% w/w). Gastrodin was quantified using a validated HPLC-PDA method with a SunFire C18 column (5 micrometers, 250 × 4.6 mm), a 10-microliter injection volume, a column temperature of 35 degrees C, a flow rate of 1.0 mL/min, and detection at 220 nm. The mobile phases were water containing 0.1% phosphoric acid and acetonitrile. The method showed acceptable specificity, a gastrodin retention time of approximately 9.6 min, linearity from 0.00725 to 0.116 mg/mL (R2 = 0.9999), recovery of 98.49−100.37%, repeatability RSD of 0.616−1.019%, a limit of detection of 0.0000408 mg/mL, and a limit of quantification of 0.0001235 mg/mL. Each 900 mg active tablet contained 500 mg of *G. elata* extract from batch N230221. The gastrodin content of this batch was 34.36 mg/g; the individual replicate results were 34.55, 34.33, and 34.20 mg/g. Two additional batches, N230224 and N230227, contained 32.28 and 33.27 mg/g gastrodin, respectively, and may be reported as batch-consistency information. (**A**) Chromatogram of the standard substance. (**B**) Chromatogram of the *G. elata* Blume, analyzed three times. (**C**) Spectrum of the standard substance. (**D**) Spectrum of *G. elata* Blume.

Gastrodin is an important bioactive component in the dried tuber of *G. elata* and has antioxidant, anti-inflammatory, anti-apoptotic and antiviral properties. This component is widely used to treat vascular and neurological diseases [30]. Effectiveness was observed at doses of 500–1000 mg/kg/day in animal studies using rats. Guidance was provided from the Center for Drug Evaluation and Research under the U.S. FDA for applying the findings from the animal study to the humans. The administered dose was 500 mg/day of *G. elata* extract in one 900 mg tablet. The animal-derived human-equivalent calculation was not used as a direct dose-matching basis because it yielded a substantially higher raw-material dose. Instead, 500 mg/day was selected as a pragmatic dose for a functional-food intervention, informed by the available human experience and feasibility of sustained daily administration The corresponding daily gastrodin exposure was approximately 17.18 mg/day based on the clinical-batch certificate of analysis. The *G. elata* tablet comprised 55.6% *G. elata* extract; 30.5% microcrystalline cellulose; 5% dextrin; 1.5% calcium carboxymethylcellulose; 1% each magnesium stearate, silicon dioxide, and hydroxypropyl methylcellulose; 0.1% glycerin; 3% white coating raw material; 0.6% Gardenia yellow pigment; 0.4% caramel pigment; 0.1% cocoa pigment; and 0.2% sodium carboxymethylcellulose. Representative HPLC-PDA chromatograms and UV spectra of the gastrodin reference standard and the *G. elata* extract are presented in Figure 2.

#### 4.4.2. Placebo Tablet Group

The placebo tablet was identical in appearance, weight (900 mg/day), and administration schedule (once daily, orally after breakfast) to the *G. elata* extract tablet but contained no *G. elata* extract. The placebo tablet comprised 55.6% dextrin, 34.5% microcrystalline cellulose, 1.5% calcium carboxymethylcellulose, 2.0% magnesium stearate, 1.0% silicon dioxide, 1.0% hydroxypropyl methylcellulose, 0.2% glycerin, 3.0% white coating raw material, 0.6% Gardenia yellow pigment, 0.4% caramel pigment, 0.1% cocoa pigment, and 0.2% sodium carboxymethylcellulose. Although most excipients were matched between the two tablets, magnesium stearate (2.0% vs. 1.0%) and glycerin (0.2% vs. 0.1%) differed slightly between the placebo and active tablet, respectively.

### 4.5. Concomitant Therapies

Concomitant medications initiated at least one month before study enrollment were permissible at the principal investigator’s discretion, provided they were judged unlikely to confound the interpretation of study outcomes. Temporary pharmacological interventions required for the management of intercurrent illnesses or emergent AEs were permitted following consultation with the attending physician. Unauthorized medication use could potentially compromise efficacy evaluation necessitated participant withdrawal from the study. Prohibited substances encompassed a broad spectrum of agents, including memory or cognition influencing health supplements, anxiolytics, antipsychotics, neurodegenerative disease medications, nootropics, neurotonics, tricyclic antidepressants, and systemic estrogen replacement therapy. All concomitant medications, their indications, and corresponding justifications were comprehensively documented in the case report form and endorsed by the principal investigator.

### 4.6. Outcomes

A case report form was used by the co-researcher and physicians at the hospital prior to data collection. A regular site visit was performed by the sponsor to verify data accuracy and completeness, for monitoring of the protocol compliance, and for observation of AEs. Cognitive and biomarker outcomes were assessed at baseline and week 12 only. No intermediate cognitive assessment was conducted at week 6. The week-6 visit was used to review medications and medical status, measure vital signs, recover and count unused tablets, assess adherence, re-dispense the study product, reinforce administration instructions, and monitor adverse events. Because identical cognitive instruments were repeated at week 12, practice effects may have contributed to improvement in both groups and cannot be separated from treatment effects. Variables used to measure efficacy were expressed as mean values, with the primary analysis comparing changes between groups after 12 weeks of intake. Furthermore, for each variable, within-group comparisons were conducted before and after intake.

The prespecified primary outcome was the change in MoCA-K total score from baseline to week 12. MoCA-K subdomains, Digit Span measures, K-MMSE outcomes, BDNF, and IL-6 were secondary outcomes. No adjustment for multiple comparisons was prespecified or applied; therefore, all secondary outcome findings are exploratory and should be interpreted cautiously.

#### 4.6.1. Primary Outcome: MoCA-K

MoCA-K was used as a primary screening tool for mild cognitive impairment [31]. The MoCA-K incorporates frontal lobe and executive function assessment items, making it particularly useful for detecting mild cognitive impairment and early-stage dementia [32]. The assessment evaluates seven cognitive domains: visuospatial/executive (5 points), naming (3 points), attention (6 points), language (3 points), abstraction (2 points), delayed recall (5 points), and orientation (6 points). The test can be administered in approximately 10–15 min, with a maximum possible score of 30 points, where higher scores indicate better cognitive performance [33].

#### 4.6.2. Secondary Outcome: Digit Span Test

The computerized instrument was the Digit Span Test included in CNT4.0L (Max Medica) [34]. Digit Span Forward used three sequences at each length from three to eight digits, and Digit Span Backward used three sequences at each length from two to seven digits. Digits were presented auditorily at one digit per second; staff entered each spoken response into the computerized system. The instrument was administered at baseline and week 12 only.

#### 4.6.3. Secondary Outcome: K-MMSE

K-MMSE was administered as a standardized cognitive assessment tool. This evaluation is relatively simple to administer and can be completed within approximately 5–10 min. Notably, it demonstrates minimal practice effects, making it well-suited for repeated measurements across time points to monitor cognitive changes over the course of disease progression or intervention [35]. The K-MMSE evaluates seven cognitive domains, with scores allocated as follows: orientation to time (5 points), orientation to place (5 points), registration (3 points), calculation (5 points), memory recall (3 points), language (8 points), and visuospatial skills (1 point), yielding a maximum total score of 30 points, where higher scores reflect better cognitive function.

#### 4.6.4. Secondary Outcome: Serum BDNF

Among the various neurotrophic factors used to evaluate neuronal plasticity, serum BDNF was selected as a blood-based biomarker. BDNF is expressed across multiple brain regions and plays a pivotal role in neuronal growth, development, and differentiation [36]. It also enhances neuronal survival and increases resistance to neuronal injury [37]. Of particular relevance to cognitive function, BDNF is highly expressed in the hippocampus, which is the brain region primarily responsible for memory consolidation, and is therefore closely associated with cognitive performance [38].

#### 4.6.5. Secondary Outcome: IL-6

IL-6, a multifunctional cytokine involved in regulating inflammation and immune responses [39], was assessed as an additional blood-based biomarker. IL-6 stimulates various types of cell proliferation and functions as an immunoregulatory molecule closely associated with mechanisms of tissue damage and the modulation of inflammatory cytokine responses [40]. Of specific relevance to cognitive aging, IL-6 concentrations increase with advancing age in elderly individuals experiencing cognitive decline [41], suggesting its utility as an inflammatory marker in cognitive function assessment.

For BDNF and IL-6, 5 mL of blood was collected into an serum-separating tube (SST) at baseline and week 12, allowed to clot for 30 min at room temperature, and centrifuged for 10 min at 3000 rpm (approximately 2000× *g*). Serum aliquots were transferred to coded microtubes, stored at −70 degrees C, and collected by the contract laboratory for analysis. BDNF was aliquoted in 0.2 mL portions, whereas at least 1 mL was retained for IL-6. Serum BDNF and IL-6 were analyzed at the Department of Physiology, College of Korean Medicine, Wonkwang University.

#### 4.6.6. Safety Assessment

Safety was comprehensively evaluated throughout the intervention period. All AEs, encompassing both subjective and objective symptoms, were systematically monitored and documented in medical terminology across both source documents and case report forms, regardless of their seriousness, severity, or relationship to the investigational product. For each recorded AE, the investigating clinician documented their assessment of the causal relationship between the event and administration of the investigational product in the case report form, with the criteria for causality assessment detailed in Supplementary Table S2. The action taken in response to each AE was also recorded and coded accordingly. Safety assessments comprised clinical laboratory investigations (hematology, blood chemistry, and urinalysis); vital sign measurements (blood pressure and pulse rate); anthropometric assessment (body weight); and electrocardiogram examination, all of which were conducted at baseline and at the end of the 12-week intervention period for both groups.

### 4.7. Sample Size

Sample size was determined based on the expected change in the MoCA-K score as the primary outcome. The sample-size calculation assumed mean MoCA-K changes of 1.9 points in the *G. elata* group and 0.1 points in the placebo group, a raw between-group difference of 1.8 points, and a common standard deviation of 2.4. This corresponds to a standardized effect size of 0.75. With 80% power, a two-sided alpha of 0.05, and equal allocation, 28 participants per group were required; allowing for 30% attrition yielded a target of 40 per group. The calculation formula used was as follows:

$$n_{C}=\frac{\left(1+\frac{1}{k}\right)\sigma^{2}{\left(Z_{\alpha /2}+Z_{\beta }\right)}^{2}}{d^{2}}$$

Accounting for an anticipated dropout rate of 30%, the final target sample size was set at 40 participants per group, yielding a total of 80 participants randomized in a 1:1 ratio.

### 4.8. Randomization, Allocation Concealment, and Blinding

Eligible participants were randomly assigned in a 1:1 ratio to either the *G. elata* or placebo group using a block randomization method with variable block sizes (4, 6, or 8 participants per block). Allocation concealment was maintained by having the sponsor retain custody of the randomization codes throughout the study. Neither the Clinical Research Coordinator, statistician, physician, nor participants had access to group assignment information during the study period, ensuring blinded condition that minimized potential bias in data collection and outcome assessment. Unblinding was strictly prohibited until all participants had completed the study and the dataset was locked. An exception was permitted solely in emergency situations requiring knowledge of a participant’s treatment assignment, in which case the principal investigator was required to notify the sponsor promptly. All instances of unblinding were documented in the case report form and source documents, including the date, time, and reason, and a copy of the sponsor’s unblinding confirmation was retained in the source documents.

### 4.9. Statistical Analysis

The primary efficacy population was the full analysis set (FAS), defined as randomized participants who received at least one dose and had at least one post-baseline efficacy assessment. Of 80 randomized participants, four in the *G. elata* group withdrew consent and had no post-baseline efficacy assessment; thus, 76 participants (36 *G.elata* and 40 placebo) were included in the FAS. The safety set included all 80 participants who consumed at least one dose. Although baseline observation carried forward was specified for missing values, it was not applicable to participants with no post-baseline efficacy assessment, who were excluded from the FAS. Continuous outcomes were summarized as mean ± SD, and change scores were compared using independent *t* tests, with paired *t* tests for within-group changes. Between-group changes were first compared using independent *t* tests. In accordance with the protocol, outcomes showing statistically significant baseline imbalance were additionally examined by ANCOVA with the corresponding baseline outcome value as a covariate. AEs were summarized by frequency and percentage and compared between groups using chi-square test or Fisher’s exact test. Missing data were handled using baseline observation carried forward. All analyses were performed using SAS version 9.4 (SAS Institute Inc., Cary, NC, USA), for Windows, with statistical significance set at *p* < 0.05.

## 5. Conclusions

This trial provides preliminary evidence supporting the efficacy and safety of 12-week *G. elata* supplementation for memory improvement in cognitively healthy adults with SMCs. The *G. elata* group demonstrated significantly greater improvements in MoCA-K total score, MoCA-K delayed recall, K-MMSE total score, and K-MMSE memory recall compared to placebo. No serious AEs were recorded throughout the trial. In this single-center, 12-week trial, *G. elata* extract was generally well tolerated and was associated with small between-group differences in MoCA-K total score and selected recall outcomes. Ceiling effects, baseline imbalance, attrition, multiplicity, retrospective registration, and the absence of an established clinically meaningful threshold preclude definitive conclusions. Larger prospectively registered trials using sensitive cognitive outcomes are needed.

## Figures and Tables

**Table 1:** Baseline Characteristics.

Variable		*Gastrodia elata* Blume Tablet Group (*n* = 40)	Placebo Group (*n* = 40)	*p* ^(1)^
Sex	Male	6	9	0.390 ^†^
	Female	34	31	
Age, years		36.50 ± 7.01	34.60 ± 7.90	0.259
Education, years		14.93 ± 1.95	14.73 ± 1.93	0.647
Height, cm		163.55 ± 6.61	166.03 ± 7.65	0.126
Weight, kg		63.94 ± 14.53	66.75 ± 17.13	0.432
Body mass index, kg/m^2^		23.73 ± 4.16	23.96 ± 4.31	0.805
Blood Pressure	Systolic	115.78 ± 12.50	115.65 ± 11.62	0.963
	Diastolic	71.20 ± 10.89	69.20 ± 8.92	0.372
	Pulse	74.28 ± 6.39	76.43 ± 8.43	0.203
Alcohol	*n* (%)	23 (57.50)	17 (42.50)	0.180 ^†^
	Units/week	1.17 ± 0.72	1.65 ± 1.29	0.180
Smoking	*n* (%)	0 (0.00)	4 (10.00)	0.116 ^#^
	Cigarettes/week	–	12.50 ± 2.89	–

Values are presented as the mean ± SD or number (%); ^(1)^ Analyzed by independent *t*-test between groups; ^†^, Analyzed by chi-square test between groups; ^#^, Analyzed by Fisher’s exact test between groups.

**Table 2:** Change in MoCA-K scores, Digit span test, K-MMSE scores, BDNF, and *IL*-6.

Efficacy Evaluation		*G. elata* Tablet Group (*n* = 36)				Placebo Group (*n* = 40)				*p* ^(2)^
		Baseline	12 Week	Change	*p* ^(1)^	Baseline	12 Week	Change	*p* ^(1)^	
MoCA-K										
Total score		27.86 ± 1.40	28.58 ± 1.13	0.72 ± 1.34	0.003 *	28.25 ± 1.33	28.38 ± 1.27	0.13 ± 1.07	0.463	0.034 *
Sub-score										
Visuospatial/executive		3.92 ± 0.84	3.94 ± 0.98	0.03 ± 0.81	0.838	3.78 ± 1.10	3.93 ± 0.97	0.15 ± 0.83	0.262	0.520
Naming		2.97 ± 0.17	3.00 ± 0.00	0.03 ± 0.17	0.324	3.00 ± 0.00	3.00 ± 0.00	0.00 ± 0.00	–	0.324
Attention		6.00 ± 0.00	6.00 ± 0.00	0.00 ± 0.00	–	6.00 ± 0.00	6.00 ± 0.00	0.00 ± 0.00	–	–
Language		2.97 ± 0.17	3.00 ± 0.00	0.03 ± 0.17	0.324	2.98 ± 0.16	3.00 ± 0.00	0.03 ± 0.16	0.324	0.941
Abstraction		2.00 ± 0.00	2.00 ± 0.00	0.00 ± 0.00	–	2.00 ± 0.00	2.00 ± 0.00	0.00 ± 0.00	–	–
Delayed Recall		4.00 ± 0.99	4.64 ± 0.59	0.64 ± 1.10	0.001 *	4.50 ± 0.91	4.40 ± 0.81	−0.10 ± 0.96	0.512	0.0003 ^†^*
Orientation		6.00 ± 0.00	6.00 ± 0.00	0.00 ± 0.00	–	6.00 ± 0.00	6.00 ± 0.00	0.00 ± 0.00	–	–
Digit Span Test	Forward digits	7.54 ± 0.98	7.88 ± 0.63	0.34 ± 0.83	0.020 *	7.93 ± 0.56	8.06 ± 0.44	0.13 ± 0.54	0.139	0.132 ^†^
	Backward digits	5.70 ± 1.10	5.99 ± 0.99	0.29 ± 1.11	0.129	6.08 ± 0.87	6.41 ± 0.92	0.32 ± 1.13	0.079	0.888
K-MMSE										
Total Score		28.50 ± 1.32	29.44 ± 0.65	0.94 ± 1.33	0.000 *	29.10 ± 0.96	29.38 ± 0.67	0.28 ± 1.04	0.102	0.003 ^†^*
Sub-score	Orientation to time	5.00 ± 0.00	5.00 ± 0.00	0.00 ± 0.00	–	4.98 ± 0.16	5.00 ± 0.00	0.03 ± 0.16	0.324	0.324
	Orientation to place	5.00 ± 0.00	5.00 ± 0.00	0.00 ± 0.00	–	5.00 ± 0.00	5.00 ± 0.00	0.00 ± 0.00	–	–
	Registration	3.00 ± 0.00	3.00 ± 0.00	0.00 ± 0.00	–	3.00 ± 0.00	3.00 ± 0.00	0.00 ± 0.00	–	–
	Calculation	4.97 ± 0.17	5.00 ± 0.00	0.03 ± 0.17	0.324	5.00 ± 0.00	5.00 ± 0.00	0.00 ± 0.00	–	0.324
	Memory recall	2.06 ± 1.04	2.78 ± 0.54	0.72 ± 1.09	0.000 *	2.60 ± 0.74	2.83 ± 0.45	0.23 ± 0.73	0.060	0.004 ^†^*
	Language	8.00 ± 0.00	8.00 ± 0.00	0.00 ± 0.00	–	7.98 ± 0.16	8.00 ± 0.00	0.03 ± 0.16	0.324	0.324
	Visuospatial	0.47 ± 0.51	0.67 ± 0.48	0.19 ± 0.67	0.090	0.55 ± 0.50	0.55 ± 0.50	0.00 ± 0.55	1.000	0.170
BDNF (pg/mL)		38,676.53 ± 8652.29	40,016.67 ± 8337.34	1340.14 ± 5772.26	0.172	39,820.50 ± 9031.21	37,622.18 ± 10,233.15	−2198.33 ± 7595.92	0.075	0.026 *
IL-6 (pg/mL)		1.11 ± 0.94	1.01 ± 0.70	−0.10 ± 0.71	0.398	1.69 ± 2.56	1.70 ± 1.87	0.01 ± 1.61	0.968	0.694

Values are presented as mean ± SD. BDNF, brain-derived neurotrophic factor; *G. elata*, *Gastrodia elata* Blume; IL-6, interleukin-6; K-MMSE, Korean Mini-Mental State Examination; MoCA-K, Korean version of the Montreal Cognitive Assessment. * *p* < 0.05 was considered statistically significant. ^(1)^ Analyzed by paired *t*-test between baseline and 12 weeks within each group. ^(2)^ Analyzed by independent *t*-test for change value between groups. ^†^ ANCOVA adjusted for the corresponding baseline outcome value; applied only to outcomes with a statistically significant baseline imbalance. All unmarked between-group *p* values were obtained from independent *t* tests of change scores. FAS, full analysis set; BDNF, brain-derived neurotrophic factor; IL-6, interleukin-6; K-MMSE, Korean Mini-Mental State Examination; MoCA-K, Korean version of the Montreal Cognitive Assessment.

**Table 3:** Study Assessment Schedule and Procedures.

Period Visit Week		Screening Visit (Week −4)	Duration of Intake		
			First Visit (Week 0)	Second Visit (Week 6)	Third Visit (Week 12)
Interventions (tablet)	*Gastrodia elata*		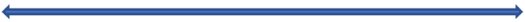		
	Placebo		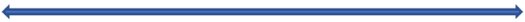		
Informed consent form		O			
Demographic information		O			
Medical history		O			
Medication history		O	O	O	O
Medical condition changes			O		
Alcohol/smoking history		O			O
Physical examination		O			O
Vital signs		O	O	O	O
Anthropometric measurements *		O			O
Subjective Memory Complaints Questionnaire		O			
Inclusion/exclusion criteria assessment		O	O		
Laboratory tests **		O			O
Electrocardiogram		O			O
Pregnancy test		O			O
MoCA-K			O		O
Digit span test			O		O
K-MMSE			O		O
Adult ADHD Self Report Scale		O			
Serum BDNF			O		O
Interleukin-6			O		O
Dietary intake and physical activity			O		O
Randomization			O		
Study product dispensation			O	O	
Adverse event monitoring				O	O
Compliance assessment				O	O

ADHD, attention-deficit/hyperactivity disorder; ASRS, Adult ADHD Self-Report Scale; BDNF, brain-derived neurotrophic factor; ECG, electrocardiography; IL-6, interleukin-6; K-MMSE, Korean Mini-Mental State Examination; MoCA-K, Korean version of the Montreal Cognitive Assessment; SMCQ, Subjective Memory Complaints Questionnaire. * Anthropometric measurements: height in cm; weight in kg; body mass index kg/m^2^ ** Laboratory tests: ALP, alkaline phosphatase; ALT, alanine aminotransferase; AST, aspartate aminotransferase; BUN, blood urea nitrogen; CK, creatine kinase; eGFR, estimated glomerular filtration rate; gamma-GT, gamma-glutamyl transferase; hCG, human chorionic gonadotropin; HbA1c, glycated hemoglobin; HDL-C, high-density lipoprotein cholesterol; hs-CRP, high-sensitivity C-reactive protein; LDH, lactate dehydrogenase; LDL-C, low-density lipoprotein cholesterol; MCH, mean corpuscular hemoglobin; MCHC, mean corpuscular hemoglobin concentration; MCV, mean corpuscular volume; RBC, red blood cell; TSH, thyroid-stimulating hormone; WBC, white blood cell. 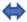 indicate the 12-week intervention period, during which the study product (*G. elata* or placebo tablet) was administered orally once daily.

## Data Availability

The data presented in this study are available on request from the corresponding author due to privacy and ethical restrictions.

## Supplementary Materials

The following supporting information can be downloaded at https://www.mdpi.com/article/10.3390/ph19101558/s1, Supplementary Table S1. Laboratory test results (safety set, *n* = 80); Supplementary Table S2. Criteria for assessing the severity of adverse events and causality of investigational products.
