# Supplementary material for: A 12-Week, Randomized, Double-Blind, Placebo-Controlled Clinical Trial of Gastrodia elata Extract for Memory Support in Adults with Subjective Memory Complaints
Source: Pharmaceuticals (Basel). 2026 Oct 1;19(10):1558. doi: 10.3390/ph19101558 (PMC13708370; doi:10.3390/ph19101558)
Supplement: Supplementary file 1 [file pharmaceuticals-19-01558-s001.zip › pharmaceuticals-4479367-supplementary.pdf]

Supplementary Material

Supplementary Table S1. Laboratory test results (safety set, n = 80).

Parameter	*Gastrodia elata* extract tablet group (n = 40)				Placebo group (n = 40)				p-value[2]
	Baseline	12 Week	Change	p-value[1]	Baseline	12 Week	Change	p-value[1]	
**CBC**									
**WBC (×10³/μL)**	5.49 ± 1.49	5.34 ± 1.10	−0.16 ± 1.35	0.474	5.65 ± 1.38	5.89 ± 1.53	0.24 ± 1.41	0.288	0.205
**RBC (×10⁶/μL)**	4.34 ± 0.38	4.38 ± 0.36	0.03 ± 0.33	0.516	4.53 ± 0.52	4.66 ± 0.45	0.13 ± 0.22	0.000	0.126
**Hemoglobin (g/dL)**	13.00 ± 1.57	12.92 ± 1.40	−0.08 ± 0.89	0.573	13.16 ± 1.70	13.47 ± 1.58	0.31 ± 0.50	0.000	0.020*
**Hematocrit (%)**	39.34 ± 4.12	39.67 ± 3.61	0.33 ± 2.69	0.439	40.15 ± 4.52	41.20 ± 4.06	1.05 ± 1.58	0.000	0.151
**MCV (fL)**	89.25 ± 9.27	90.63 ± 4.69	1.38 ± 8.27	0.297	88.88 ± 5.16	88.61 ± 4.99	−0.26 ± 1.44	0.252	0.222
**MCH (pg)**	29.89 ± 1.98	29.51 ± 2.06	−0.38 ± 0.80	0.005	29.08 ± 2.06	28.94 ± 2.08	−0.14 ± 0.65	0.173	0.155
**MCHC (g/dL)**	33.02 ± 1.07	32.53 ± 0.93	−0.49 ± 0.62	<0.0001	32.72 ± 0.91	32.65 ± 0.87	−0.07 ± 0.61	0.470	0.003*
**Platelet (×10³/μL)**	252.20 ± 64.44	256.33 ± 59.81	4.13 ± 32.14	0.422	263.18 ± 46.94	263.00 ± 51.19	−0.18 ± 29.92	0.971	0.538
**Biochemistry**									
**Total protein (g/dL)**	7.12 ± 0.40	7.06 ± 0.32	−0.06 ± 0.38	0.339	7.15 ± 0.31	7.11 ± 0.35	−0.03 ± 0.28	0.469	0.737
**Albumin (g/dL)**	4.37 ± 0.25	4.39 ± 0.25	0.02 ± 0.21	0.597	4.38 ± 0.23	4.40 ± 0.23	0.02 ± 0.20	0.580	1.000
**Total bilirubin (mg/dL)**	0.72 ± 0.24	0.76 ± 0.33	0.05 ± 0.25	0.256	0.77 ± 0.28	0.74 ± 0.29	−0.03 ± 0.33	0.570	0.255
**ALP (IU/L)**	156.20 ± 41.20	150.65 ± 39.03	−5.55 ± 21.25	0.107	162.53 ± 41.01	158.15 ± 43.75	−4.38 ± 22.16	0.219	0.809
**AST (IU/L)**	22.78 ± 6.40	24.48 ± 10.12	1.70 ± 7.26	0.147	21.80 ± 5.35	23.50 ± 9.95	1.70 ± 7.70	0.170	1.000
**ALT (IU/L)**	18.93 ± 12.75	21.38 ± 21.09	2.45 ± 10.09	0.133	19.18 ± 10.45	20.98 ± 18.67	1.80 ± 11.22	0.316	0.786
**LDH (U/L)**	172.03 ± 47.96	174.73 ± 35.50	2.70 ± 51.85	0.744	162.15 ± 30.25	177.75 ± 43.77	15.60 ± 41.18	0.022	0.222
**Gamma-GT (IU/L)**	17.63 ± 8.82	17.85 ± 12.05	0.23 ± 6.87	0.837	20.78 ± 13.31	18.65 ± 9.85	−2.13 ± 7.86	0.095	0.159
**Glucose (mg/dL)**	81.98 ± 8.65	84.60 ± 9.63	2.63 ± 10.97	0.138	78.85 ± 10.56	82.23 ± 10.36	3.38 ± 13.07	0.110	0.782
**HbA1c (%)**	5.42 ± 0.35	5.56 ± 0.33	0.14 ± 0.17	<0.0001	5.39 ± 0.35	5.57 ± 0.33	0.18 ± 0.16	<0.0001	0.279
**BUN (mg/dL)**	11.47 ± 2.88	11.80 ± 2.77	0.33 ± 2.00	0.303	11.31 ± 2.13	11.13 ± 2.71	−0.17 ± 2.89	0.708	0.369
**Creatinine (mg/dL)**	0.77 ± 0.14	0.81 ± 0.16	0.04 ± 0.08	0.003	0.76 ± 0.13	0.80 ± 0.16	0.05 ± 0.08	0.001	0.826
**eGFR (mL/min/1.73 m²)**	105.63 ± 12.71	101.03 ± 13.36	−4.60 ± 8.71	0.002	109.60 ± 9.66	104.85 ± 10.96	−4.75 ± 9.47	0.003	0.941
**Total cholesterol (mg/dL)**	191.85 ± 35.62	201.40 ± 34.05	9.55 ± 24.29	0.017	186.25 ± 26.30	192.58 ± 26.99	6.33 ± 22.39	0.082	0.539
**HDL-C (mg/dL)**	61.40 ± 11.83	62.35 ± 11.03	0.95 ± 7.29	0.415	59.95 ± 15.79	60.38 ± 15.04	0.43 ± 8.82	0.762	0.772
**LDL-C (mg/dL)**	108.28 ± 33.75	114.68 ± 31.57	6.40 ± 20.68	0.058	107.20 ± 27.14	109.40 ± 28.37	2.20 ± 13.75	0.318	0.289
**Triglyceride (mg/dL)**	85.58 ± 50.42	88.80 ± 49.99	3.23 ± 41.15	0.623	80.95 ± 57.95	95.85 ± 89.01	14.90 ± 63.99	0.149	0.335
**CK (U/L)**	84.10 ± 60.02	109.65 ± 98.23	25.55 ± 67.95	0.022	86.08 ± 46.15	104.10 ± 81.20	18.03 ± 75.32	0.138	0.640

**hs-CRP (mg/L)**	0.85 ± 1.42	0.63 ± 0.78	−0.22 ± 1.40	0.320	0.87 ± 1.19	1.28 ± 2.16	0.41 ± 1.64	0.121	0.066
**TSH (μIU/mL)**	1.61 ± 0.75	1.90 ± 1.33	0.29 ± 1.19	0.134	1.52 ± 0.68	1.77 ± 0.91	0.25 ± 0.87	0.074	0.877
**Urinalysis**									
**pH**	6.45 ± 1.07	6.44 ± 1.03	−0.01 ± 0.92	0.932	6.54 ± 1.00	6.53 ± 0.94	−0.01 ± 1.15	0.945	1.000
**Specific gravity**	1.02 ± 0.01	1.02 ± 0.01	0.00 ± 0.01	0.324	1.02 ± 0.01	1.02 ± 0.01	0.00 ± 0.01	0.529	0.262

ALP, alkaline phosphatase; ALT, alanine aminotransferase; AST, aspartate aminotransferase; BUN, blood urea nitrogen; CK, creatine kinase; eGFR, estimated glomerular filtration rate; Gamma-GT, gamma-glutamyl transferase; HbA1c, glycated hemoglobin; HDL-C, high-density lipoprotein cholesterol; hs-CRP, high-sensitivity C-reactive protein; LDH, lactate dehydrogenase; LDL-C, low-density lipoprotein cholesterol; MCH, mean corpuscular hemoglobin; MCHC, mean corpuscular hemoglobin concentration; MCV, mean corpuscular volume; RBC, red blood cell; TSH, thyroid-stimulating hormone; WBC, white blood cell.

Values are presented as mean ± SD.

* $p < 0.05$ was considered significant; significant values are highlighted in red.

[1] Analyzed by paired *t*-test between baseline and 12 weeks within each group.

[2] Analyzed by independent *t*-test for change value between groups.

Supplementary Table S2. Criteria for assessing the severity of adverse events and causality of investigational products.

**Adverse Event Severity Grades**	
**Grade**	**Definition**
1 = Mild	Does not interfere with the subject's normal daily activities (functioning) to an extent that the subject barely notices. Treatment is generally not required.
2 = Moderate	May cause discomfort to the subject and interferes with normal daily activities (functioning). The subject is able to continue participation in the trial, but treatment may be required.
3 = Severe	Causes significant discomfort to the subject, rendering normal daily activities (functioning) impossible and making continued participation in the trial unfeasible. Medical treatment or hospitalization may be required.
**Causality Assessment**	
**Causality**	**Criteria**
Definitely related	There is evidence that the subject consumed the investigational product; consumption of the investigational product provides the most plausible explanation above all other causes; the adverse event resolves upon discontinuation of intake; rechallenge result (conducted only where possible) is positive; the adverse event is consistent with information already known regarding the investigational product or foods of the same class.
Probably related	There is evidence that the subject consumed the investigational product; the temporal sequence between consumption of the investigational product and onset of the adverse event is plausible; the adverse event is more plausibly attributable to consumption of the investigational product than to any other cause; the adverse event resolves upon discontinuation of intake.
Possibly related	There is evidence that the subject consumed the investigational product; the temporal sequence between consumption of the investigational product and the onset of the adverse event is plausible; the adverse event is considered equally attributable to the investigational product as to other possible causes; the adverse event resolves upon discontinuation of intake (where conducted).
Probably not related	There is no evidence that the subject consumed the investigational product; there is a more plausible alternative cause for the adverse event; the result of discontinuation (where conducted) is negative or equivocal.
Definitely not related	There is no evidence that the subject consumed the investigational product; there is another cause that most plausibly explains the adverse event; the adverse event does not resolve upon discontinuation of intake (where conducted).
Unknown	The information is insufficient or contradictory such that a judgment cannot be made, and this cannot be supplemented or confirmed.
